# Editorial for Special Issue ‘Engineering and Characterisation of Novel Nanomedicine Formulations’

**DOI:** 10.3390/pharmaceutics16050585

**Published:** 2024-04-26

**Authors:** Raquel Fernández-García, Francisco Bolás-Fernández, Ana Isabel Fraguas-Sánchez

**Affiliations:** 1 School of Pharmacy, University of Nottingham, University Park, Nottingham NG7 2RD, UK; 2 Department of Microbiology and Parasitology, Faculty of Pharmacy, Complutense University, 28040 Madrid, Spain; francisb@ucm.es; 3 Department of Pharmaceutics and Food Technology, Faculty of Pharmacy, Complutense University, 28040 Madrid, Spain; 4 Institute of Industrial Pharmacy, Complutense University, 28040 Madrid, Spain

Nanomedicine is the application of nanotechnology to achieve innovations in healthcare and involves the engineering of systems at the nanoscale (particle size < 1000 nm) with the aim of improving drug delivery. These systems can modify the release profile of drugs, but also encapsulate poorly soluble drugs and modify their biodistribution, thus achieving the selective targeting of tissues and minimising adverse effects. The encapsulation of drugs within nanosystems also protects the drug from degradation, with it being suitable for sensitive substances, such as proteins or RNA.

This Special Issue entitled ‘Engineering and Characterisation of Novel Nanomedicine Formulations’ highlights the latest findings in the development of these systems. We are deeply grateful for the 13 articles covering a variety of interesting achievements. The articles cover multiple nanosystems, including nanoparticles (polymeric, lipid and magnetic), ethosomes, liposomes, exosomes, dendrimers and metal complexes.

It is well known that lipid formulations have demonstrated their usefulness in increasing drug permeation through the skin [1]. Rarokar et al. (contribution 1) developed solid lipid nanoparticles (SLNs) loaded with terbinafin hydrochloride, an antifungal drug with a high hepatic first pass metabolism and oral bioavailability of 30–40% [2]. These nanoparticles were incorporated into a carbopol-based hydrogel for their topical application. This hydrogel showed a better skin deposition and higher in vitro antifungal activity than marketed formulations of this drug. Huanbutta et al. (contribution 2) developed ethosomes as a strategy to improve the permeation of Zingiber zerumbet Linn Rhizome Extract that has demonstrated its antifungal properties, for example, against *Candida albicans.* Its encapsulation significantly enhanced the permeation and retention compared to the non-encapsulated extract.

Lipid nanoparticles have also been shown to be useful in delivering oligonucleotides, including siRNAs, due to their high encapsulation efficiency, improved circulation times and reduced immunogenicity [3]. siRNAs are being exploited as therapeutics in a broad range of conditions, including cancer disease [4]. They are highly useful in the treatment of acute myeloid leukaemia. Most haematological diseases, including leukaemia, tend to originate in the bone marrow. In this context, lipid nanoparticles encapsulating siRNAs and accumulated in this tissue could represent a promising nanoformulation for the treatment of this neoplasm. To achieve this selective accumulation in the bone marrow, Swart et al. (contribution 3) developed lipid nanoparticles targeting very late antigen-4 (VLA-4), which is highly expressed in the bone marrow [5]. These researchers prepared lipid nanoparticles functionalised with the tripeptide Leu-Asp-Val, which binds VLA-4 receptors. The functionalised formulation showed a higher accumulation and retention in the bone marrow than their counterparts, resulting in an excellent strategy for improving the treatment of leukaemia.

It is well known that the use of nanocarriers is also an excellent strategy to increase the efficacy and decrease the adverse effects of antineoplastics in solid tumours [6]. Nanoformulations facilitate targeted drug localisation, specifically at the tumour site. For example, Rocha-Gomes et al. (contribution 4) developed pH-sensitive liposomes encapsulating doxorubicin to improve the efficacy of this drug in triple-negative breast cancer, a difficult-to-treat and invasive carcinoma, the treatment of which remains an unmet need worldwide. This formulation exhibited a lower toxicity compared to free drugs. Moreover, it exhibited a tumour-suppressive effect and a lower incidence of metastatic foci in the lungs in a 4T1 breast cancer model developed in mice. On the other hand, it has been demonstrated that the hypoxic tumour microenvironment contributes to the appearance of resistances and metastases and, consequently, to a poor prognosis. Bhatavdekar et al. (contribution 5) demonstrated that nanomedicine could help to overcome this complication. They showed that pH-sensitive nanoparticles loaded with cisplatin allow for a fast and uniform exposure of tumour cells to this drug, inhibiting the population of hypoxia-affected cells and their metastatic potential. The use of nanoencapsulated cisplatin was more effective than free drugs.

It should be noted that nanocarriers also offer the capability to encapsulate multiple drugs simultaneously. This presents a compelling prospect in tumour therapy, as it enables the coordinated release of these agents in the tumour microenvironment, potentially augmenting their synergistic therapeutic effects. Duarte et al. (contribution 6) used this strategy. These researchers developed pH-sensitive liposomes encapsulating both simvastatin and doxorubicin. In TNBC, a higher cytotoxic effect was observed with this formulation compared to the administration of the free drugs in combination. For example, while the combination of doxorubicin and simvastatin at molar ratios of 1:1 and 1:2 showed IC50 values of around 0.96 and 0.71 µM, respectively, the nanoencapsulated drugs showed values of around 0.47 and 0.35 µM at the same ratios, respectively.

Tang et al. (contribution 7) evaluated the efficacy of nanoencapsulated doxorubicin in lung cancer, which is the leading cause of cancer death worldwide [7]. They developed nanoformulations using laponite and linear or cyclic poly(ethylene glycol) (PEG). However, these nanosystems did not exert a significantly higher antiproliferative effect in A549 than free doxorubicin. This cytotoxic effect washigher in the formulations developed with cyclic poly(ethylene glycol). Moreover, these formulations also exerted a higher apoptotic activity in primary lung epithelial cells. These results suggest that nanosystems elaborated with laponite and cyclic-PEG could represent an interesting strategy to administer doxorubicin in cancer therapy.

Garcés-Garcés et al. (contribution 8) synthesised a novel group of silver and copper complexes based on perylenediimide that showed an antiproliferative effect in cervical cancer cells (HeLa cells). While the free perylenediimide ligands displayed moderate cytotoxicity, coordination with silver or copper notably augmented the activity, suggesting a synergistic interplay between these compounds.

Vaccine development is another therapeutic area where nanoparticles show a great potential. For example, Xia et al. (contribution 9) developed S60-VP8* pseudovirus nanoparticles as a parenteral vaccine candidate for the most prevalent rotavirus infections. It was a trivalent vaccine with the glycan receptor binding VP8* domains of rotavirus spike proteins, and it exerted a high and broad immunogenicity in mice when administered intramuscularly, resulting in a promising candidate for this disease that causes vomiting, severe watery diarrhoea, abdominal pain and/or fever usually in infants and young children.

It is imperative to evaluate the potential toxicity and interaction with biological structures of nanocarriers [8]. For example, magnetic nanoparticles (MNPs) exhibit exceptional characteristics, rendering them suitable for application as therapeutic agents in hyperthermia, as well as in adjuvant local anticancer therapy that involves elevating temperatures beyond the physiologically optimal range, typically from 40 to 43 °C. In this context, it is essential to study the interaction mechanisms experienced by MNPs upon intravenous administration, as upon introduction into the bloodstream, MNPs transition from a synthetic identity to a biological one. The study by Marassi et al. (contribution 10) aimed to study this aspect. These researchers employed a dynamic methodology utilising flow field-flow fractionation and showed that, within a dynamic biological environment and subsequent to interactions with serum albumin, MNPs maintain their colloidal properties. This supports their safety profile for intravenous administration and use in hyperthermia.

Dendrimers are nanoscale molecules characterised by symmetrical structures, wherein a central atom or group of atoms is enveloped by branching units referred to as dendrons with potential application as drug delivery carriers [9]. Akhtar et al. (contribution 11) evaluated the impact of physiochemical attributes of dendrimer nanoparticles on cardiac contractility and hemodynamics. Specifically, these researchers examined the influence of polyamidoamine (PAMAM) dendrimer generation (G7, G6, G5, G4 and G3) and surface chemistry (–NH2, –COOH and –OH). This study revealed that cardiac function impairment diminished with decreasing dendrimer generation, with G3 exhibiting minimal or no cardiotoxicity. Cationic PAMAMs (–NH2) were more toxic than anionic (–COOH), while neutral PAMAMs (–OH) displayed the least cardiotoxicity. Cationic G7 PAMAM-induced cardiac dysfunction was significantly ameliorated by Ang-(1-7) administration.

As discussed by Dri et al. (contribution 12), although nanoparticulate technology has revolutionised the treatment of many pathologies such as infectious disease, cancer and neurological disorders, among others, the clinical translation of nanomedicines is extremely complex. One of the main challenges is to find elaboration methods that can be applied on a large scale and allow for the production of nanomedicines that preserve their original features and show a low batch-to-batch variability. In this context, microfluidics has emerged as a new and excellent strategy for the industrial manufacturing of nanomedicines. This strategy allows for the obtention of highly tuneable and reproducible strategies. All these aspects were discussed by Osouli-Bostanabad et al. (contribution 13) in their review paper.
